# FemtoMAX – an X-ray beamline for structural dynamics at the short-pulse facility of MAX IV

**DOI:** 10.1107/S1600577517017660

**Published:** 2018-02-14

**Authors:** Henrik Enquist, Andrius Jurgilaitis, Amelie Jarnac, Åsa U. J. Bengtsson, Matthias Burza, Francesca Curbis, Christian Disch, J. Carl Ekström, Maher Harb, Lennart Isaksson, Marija Kotur, David Kroon, Filip Lindau, Erik Mansten, Jesper Nygaard, Anna I. H. Persson, Van Thai Pham, Michael Rissi, Sara Thorin, Chien-Ming Tu, Erik Wallén, Xiaocui Wang, Sverker Werin, Jörgen Larsson

**Affiliations:** a MAX IV Laboratory, Lund University, PO Box 118, Lund 22100, Sweden; b Department of Physics, Lund University, PO Box 118, Lund 22100, Sweden; c Dectris AG, Taefernweg, Baden-Daettwil 15405, Switzerland; d Departments of Physics and Materials Science and Engineering, Drexel University, Philadelphia, PA 19104, USA; e Department of Environmental Science, Aarhus University, Roskilde 4000, Denmark; f Center for Quantum Electronics, Institute of Physics, Vietnam Academy of Science and Technology, Hanoi, Vietnam; g Lawrence Berkeley National Laboratory, Berkeley, CA 94720, USA

**Keywords:** structural dynamics, pump–probe, laser, beamline, ultrafast

## Abstract

The FemtoMAX beamline facilitates studies of the structural dynamics of materials. Such studies are of fundamental importance for key scientific problems related to programming materials using light, enabling new storage media and new manufacturing techniques, obtaining sustainable energy by mimicking photosynthesis, and gleaning insights into chemical and biological functional dynamics. The FemtoMAX beamline utilizes the MAX IV linear accelerator as an electron source. The photon bursts have a pulse length of 100 fs, which is on the timescale of molecular vibrations, and have wavelengths matching interatomic distances (Å). The uniqueness of the beamline has called for special beamline components. This paper presents the beamline design including ultrasensitive X-ray beam-position monitors based on thin Ce:YAG screens, efficient harmonic separators and novel timing tools.

## Abstract 2

The FemtoMAX beamline facilitates studies of the structural dynamics of materials on the femtosecond timescale. The first commissioning results are presented.

## 1. Introduction   

Investigating structural dynamics in molecular systems on timescales that are pertinent to revealing short-lived transient states is an evolving scientific field. The first major results were obtained on the 100 ps timescale at the ID09 beamline at the European Synchrotron Radiation Facility (ESRF), where dynamics of myoglobin were studied (Srajer *et al.*, 1996 ▸). Following that, short-pulse, laser-based and accelerator-based sources were developed (Rischel *et al.*, 1997 ▸; Rose-Petruck *et al.*, 1999 ▸; Chin *et al.*, 1999 ▸). Some of the research highlights using these sources were studies of acoustic phonons in semiconductors (Lindenberg *et al.*, 2000 ▸), non-thermal melting of InSb (Sokolowski-Tinten *et al.*, 2001 ▸; Rousse *et al.*, 2001 ▸) and optical phonons in Bi (Sokolowski-Tinten *et al.*, 2003 ▸). Up until 2003 there was no X-ray source that could combine the short pulses available from the laser-based sources with the low divergence generally achieved at the accelerator-based sources. However, focusing optics for laser-based sources were improved (Nicoul *et al.*, 2005 ▸) and the temporal resolution at accelerator-based sources could be improved using streak cameras (Larsson *et al.*, 1998 ▸) and slicing techniques (Schoenlein *et al.*, 2000 ▸; Ingold *et al.*, 2008 ▸).

In 2003 a major step forward for ultrafast X-ray science was achieved when the sub-picosecond photon source (SPPS) was launched at SLAC. It provided a wealth of scientific results during about ten campaigns of 2–3 week periods (Lindenberg *et al.*, 2005 ▸, 2008 ▸; Gaffney *et al.*, 2005 ▸; Cavalieri *et al.*, 2005 ▸). The SPPS was terminated in 2006 when the space was needed for the construction of the LINAC Coherent Light Source (LCLS). During the construction of LCLS, the femto-slicing beamline at the Swiss Light Source (SLS) was the leading source for time-resolved X-ray measurements in spite of the limited flux (Johnson *et al.*, 2008 ▸; Harb *et al.*, 2011 ▸; Mariager *et al.*, 2012 ▸; Caviezel *et al.*, 2012 ▸). In 2010 the completion of the X-ray pump–probe (XPP) instrument at LCLS enabled hard X-ray pump–probe studies using a free-electron laser (FEL). In the first years, XPP provided insights into the dynamics of ferroelectrics (Daranciang *et al.*, 2012 ▸), the dynamics of basic chemical processes (Haldrup *et al.*, 2016 ▸) and provided basic knowledge of structural dynamics in various materials (Trigo *et al.*, 2013 ▸). A recent overview of the XPP instrument describes the instrumentation in detail (Chollet *et al.*, 2015 ▸). FemtoMAX is an incoherent source with significantly lower flux than LCLS, but with the aim of being able to carry out a large portion of the solid-state and chemical physics science programmes that today rely on over-subscribed hard X-ray FELs.

### 1.1. Overview of SPF and FemtoMAX beamline   

**Figure 1:**
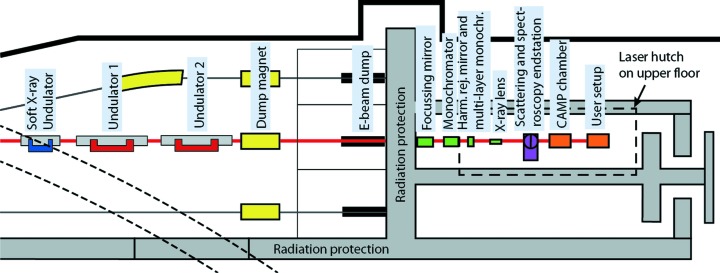
The FemtoMAX hard X-ray beamline at the MAX IV short-pulse facility.

The short-pulse facility (SPF) is driven by the linear accelerator (LINAC) at MAX IV. The design of the LINAC and SPF allows for electron bunches shorter than 100 fs in duration. We will, in the next section, briefly discuss the LINAC and in particular the properties enabling FemtoMAX. The scope of the paper is to describe the FemtoMAX beamline which, so far, is the only SPF beamline. We will give a detailed description of the FemtoMAX beamline including specifications and present status of the components shown in Fig. 1 ▸.

The FemtoMAX beamline is equipped with a 666-period undulator with 10 m active length. When the short electron pulses are sent through this insertion device they emit femtosecond X-ray pulses with photon energy from 1.8 keV and higher. The first element of the front-end is a dipole magnet that bends the electrons onto an electron dump. The X-ray beam is defined by an adjustable slit positioned 10 m after the exit of the undulator. The position and flux of the beam can be measured using an X-ray beam-position monitor (X-BPM) based on a thin Ce:YAG screen and an absolute calibrated Si diode. The front-end also contains a user shutter which can reduce the pulse-repetition rate of the X-rays and the safety shutters. Two sets of focusing optics are available. A toroidal mirror in the beamline hutch images the source with a (de)magnification of 0.4 at the end-stations. Cylindrical Be lenses can be used to vertically obtain a (de)magnification of 0.1 at the end-station. One double-crystal monochromator and one double multi-layer mirror monochromator are available. An overview of the beamline can be seen in Fig. 1 ▸. An ultrafast laser system provides femtosecond optical pulses for optical pump–X-ray probe experiments. The laser system is located in a laser laboratory directly above the beamline. The laser oscillator is synchronized to the 3 GHz radiofrequency (RF) signal originating from the master oscillator. The synchronization between laser and electrons is monitored by an optical cross-correlator and an RF filter-based jitter monitor. The direct timing between laser and X-rays can be measured by a UV-sensitive, X-ray streak camera. End-stations for scattering and spectroscopy are built to be interchangeable, whereas a chamber for atomic and molecular physics is placed behind the scattering and spectroscopy end-station. Single-photon-sensitive two-dimensional detectors including a Pilatus3 1.2M and a direct detection detector built in CMOS technology are available.

### 1.2. SPF electron bunch structure   

The SPF is an accelerator facility allowing for use of longitudinally compressed electron bunches for generation of X-ray radiation (Werin *et al.*, 2009 ▸). The MAX IV LINAC (Thorin *et al.*, 2014 ▸) is operated at 3 GeV. The electron pulses are generated in a field which is shaped to give a linear longitudinal energy spread (chirp) during initial acceleration. A longitudinally extended bunch reduces the Coulomb repulsion during acceleration. The electron bunches are longitudinally compressed to 100 fs in two bunch compressors (BC1, BC2) (Thorin *et al.*, 2010 ▸). The SPF is designed to have optimally compressed pulses for a bunch charge of 100 pC. However, it has also been operated at charges above 200 pC, at the expense of pulse duration and emittance. The MAX IV LINAC is a stable electron source, as the pulse charge varies less than 10%, and the fluctuations of position and angle of the beam fall within 10% of the beam size and divergence. The design and measured values are given in Table 1 ▸. The design of the beamline optics is dependent on the emittance and beta function of the electron source. We have used a conservative value of 10 mm mrad for the normalized emittance of the electron beam. This gives a non-normalized root mean square (RMS) value of 1.6 nm rad. The target value for the normalized emittance is 1 mm mrad and a value of 0.5 mm mrad has been measured near the photo-electron gun. A lattice which allows the beta function (describing the electron beam focus) to vary between 10 m and 15 m horizontally and vertically through the 12 m-long undulator section has been chosen. A beta function of 15 m and an emittance of 1.6 nm rad gives an electron source size of 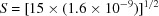 = 150 µm. The divergence is given as 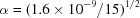 = 10 µrad.

Work to measure and minimize the bunch duration is still ongoing. With full compression in BC1, a full width at half-maximum (FWHM) bunch length of 160 fs was recorded using a screen in a dispersive section of BC2 as diagnostics. Lacking a transverse deflecting cavity, measurements of longitudinal phase space have been made using a version of the zero-crossing method. A streak of the beam was made in a dispersive section of BC2, when accelerating the beam 20° off crest in the whole main LINAC. This makes the profile along the horizontal axis on the screen proportional to the longitudinal profile of the beam..

## 2. Insertion devices   

The beamline design contains insertion devices which fit in the 15 m-long straight section of this SPF beamline. As a compromise between budget and performance, we choose a 10 m-long short-period in-vacuum undulator covering the energy range 1.85–20 keV with a continuous tuning range. The undulator is divided into two 5 m-long sections with a phase-shifter in between sections. In order to obtain the fundamental energy operating at 1.8 keV for the 3 GeV source, we choose an undulator period of 15 mm. The *K* value can be tuned between 0.5 and 2.2 which provides a complete spectral coverage without gaps. A magnetic design requiring a 2 mm undulator gap has been chosen. The undulators have been delivered by Hitachi/Neomax. A temporary long-period undulator, designed for generating soft X-rays (Denecke *et al.*, 1999 ▸), is presently installed in a third 5 m section. This is the device labelled ‘Soft X-ray Undulator’ in Fig. 1 ▸. It is a 49-period undulator with a 52 mm period and gap that is adjustable down to 22 mm (limited by SPF vacuum tube). The results presented here are obtained by this undulator as the in-vacuum undulator is not yet installed. Calculations for the undulator flux have been carried out using *SPECTRA* v10 (Tanaka & Kitamura, 2001 ▸). The source flux incident on the X-ray optics is given in Table 2 ▸.

## 3. X-ray optics   

The divergence of the undulator radiation is limited by the electron beam divergence and 1/γ, where γ is the relativistic gamma factor, and is expected to be less than 20 µrad. The unfocused beam will be less than 1 mm at the user end-stations. Because of the low average power, the X-ray optics design is simpler at the SPF than at the 3 GeV ring. The average LINAC current will be below 1 × 10^−7^ A and there is no need for cooling. Even though the very short duration of the X-ray pulses leads to a high peak power, it is still well below the thresholds for damage of optical components. The high peak energy leads to a fast temperature increase at the surface of the illuminated area. There is however a finite electron–phonon coupling time, typically a few picoseconds, meaning that no significant heat is transferred to the lattice during the 100 fs X-ray pulse. Before the next pulse arrives 10 ms later, the heat has dissipated.

### 3.1. Harmonic rejection   

The radiation produced by an undulator contains both the fundamental energy and several harmonics. A monochromator can be used to remove energies lower than the set energy. Higher energies may however still be transmitted. These have to be removed by an additional device, such as harmonic rejection mirrors. This works by the principle that the critical angle for specular reflection normally is smaller for higher photon energies.

By positioning a mirror so that the desired energy is reflected close to the critical angle, the harmonics with higher energy can be rejected. For harmonics with photon energies lower than 10 keV, harmonic suppression can be carried out using harmonic rejection mirrors. To keep the outgoing beam parallel to the incoming, a set of two carbon-coated mirrors is used in a configuration similar to a double-crystal monochromator. The main parameters for the harmonic rejection mirrors are given in Table 3 ▸. The harmonic rejection mirrors have been implemented as stripes on the multi-layer mirror monochromator substrates.

At higher energies, the harmonic separation prism will be used since it provides better throughput. The harmonic separation prism uses the dispersion of Be in order to select a single undulator harmonic using a downstream slit. The principle has been described by Burza *et al.* (2015 ▸). The prism is positioned close to the undulator after the first beam-defining slit. The dispersion results in harmonics being separated in the horizontal plane. By using a second slit, a given harmonic can be chosen and the rejection ratio can be traded-off against flux by varying the slit sizes. The energy separation for undulator harmonics is larger for a higher fundamental energy which is manifested as a larger horizontal separation and a larger useful energy range. As an example, for a third harmonic beam at 12 keV, incident on the prism at an angle of 89.5° relative to the surface normal, about 50% of the light is transmitted. The separations for the third harmonic at the plane of the mirror, 10 m downstream, is 1.2 mm from the fourth harmonic at 16 keV and 2.5 mm from the second harmonic. In this case the limiting factor that will set the rejection ratio is the scattering from inclusions and imperfections at the prism surface. The useful spectral ranges where a 0.5 mm separation can be reached together with a <50% absorption in the prism are given in Table 4 ▸

### 3.2. Monochromators   

**Figure 2:**
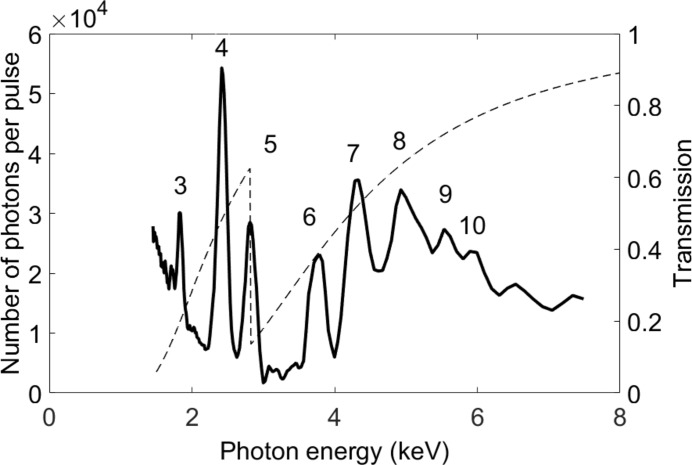
Monochromator scan using the MLM with the Ni-B_4_C stripes. The harmonic numbers are given. The beam has passed through a Parylene C filter so that the chlorine edge at 2824 eV is visible. The filter transmission (right axis) is given as a dashed line. The photon numbers correspond to the detected number of phonons after the calibration filter and the multi-layer mirror pair, which has a transmission ranging between 30% and 50% in this photon energy range.

For monochromatization, several alternatives are needed. The double-crystal monochromator (DCM) houses two sets of crystals which can be interchanged by moving a translation stage. As can be seen in Table 5 ▸, InSb (111) extends to softer X-rays and provides a wider bandwidth compared with Si (111). A multi-layer monochromator (MLM) is required to obtain the highest possible flux for wide-angle X-ray scattering (WAXS) experiments on liquids while at the same time suppressing the low-energy tail in the undulator spectrum. Three different multi-layer mirror pairs have been coated onto the same substrates together with the harmonic rejection stripe in order to conveniently optimize the performance for different wavelength ranges. The characteristics of the monochromators are shown in Table 5 ▸. A monochromator scan using the MLM and the temporary soft X-ray undulator is shown in Fig. 2 ▸. The flux from the undulator and throughput of the monochromator is within specifications for the given range.

### 3.3. Focusing optics   

**Figure 3:**
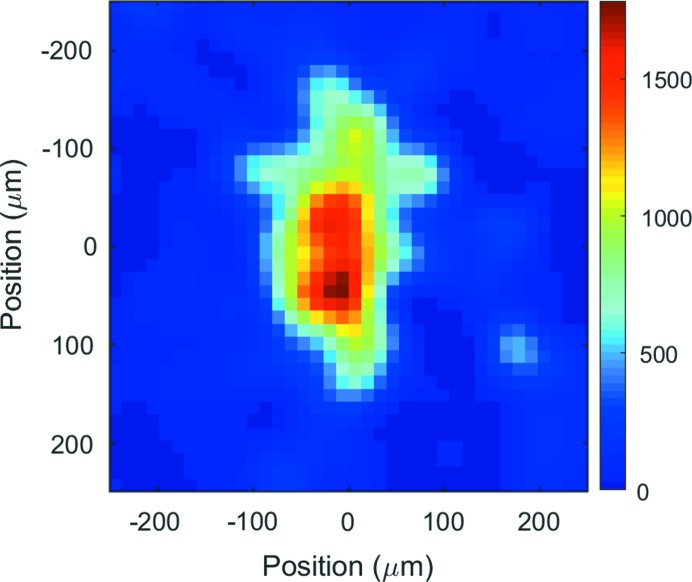
Focused beam at the position of the scattering end-station. The beam size FWHM is 80 µm × 160 µm.

A cylindrical Rh-coated Si mirror with an incidence angle of 0.14–0.18° which can be bent to a toroid has been commissioned. It has been designed to focus the radiation from both the soft X-ray undulator and the in-vacuum undulator. The focusing mirror parameters can be seen in Table 6 ▸. The in-vacuum undulator source point is 15 m before the mirror, and the focus can be positioned at all end-station locations. For the soft X-ray undulator radiation, the source distance is 30 m and the image distance is 7 m. For this case we have observed a spot size of 80 µm in the horizontal direction and 160 µm in the vertical direction at the scattering end-station. This corresponds to a (electron) source size of 0.3 mm × 0.6 mm. The measured X-ray focal spot from the soft X-ray undulator is shown in Fig. 3 ▸. The beam profile was recorded in air directly after a helium-flushed tube.

For many experiments a smaller spot, than what can be achieved with the mirror system, is needed. This can be achieved with Be lenses at the expense of the throughput. The beryllium compound refractive lenses for micro-focusing are useful for the radiation from the permanent short-period undulator. The distance from the source point at the end of the undulator to the sample holder in the goniometer is 24.85 m. A set of vertically focusing lens stacks can be inserted 2.2 m before the sample holder. A binary insertion system is used to facilitate focusing of a wide range of photon energies. The bender for the toroidal mirror is then set to flat, so that the mirror only provides horizontal focusing. This setup gives a demagnification of the source by a factor of 11. The beam size at the output of the undulator is designed to be 150 µm and the resulting X-ray spot on the sample will have a vertical size of <15 µm. Fine-tuning of the focal position can be achieved by slightly bending the focusing mirror. The range over which a throughput larger than 50% can be achieved is 2.5–20 keV for a one-dimensional focus suitable for grazing-incidence diffraction.

## 4. X-ray diagnostics   

### 4.1. X-ray beam-position monitors   

**Figure 4:**
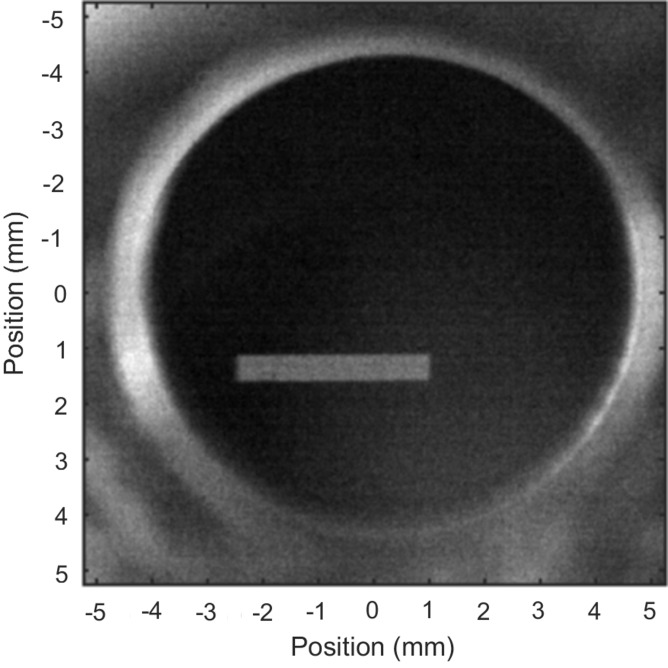
X-BPM image of the beam just after the focusing mirror. The approximately 8 mm free aperture round Ce:YAG disc can be seen.

The five X-BPMs are based on 20 µm-thick Ce-doped YAG crystals Ce:YAG. Each crystal is imaged onto an Andor ZYLA sCMOS camera. Each X-BPM is placed directly after a four-blade slit which is used for maintaining a reference position for the X-ray beam. For energies above 10 keV, the transmission is >70% and one BPM can remain in place. For softer X-rays the X-BPM can be retracted. The beam from the soft X-ray undulator on the first X-BPM after the focusing mirror is shown in Fig. 4 ▸. The beam is cut horizontally by a 5 mm-wide filter and vertically by the mirror. The 400 mm-long mirror has a vertical aperture of about 1.2 mm at an incidence angle of 0.2°. The X-BPMs are mounted on the same translation stage as calibrated large-area Si diodes (10 mm × 10 mm), so that a photon flux can be obtained at each X-BPM.

### 4.2. X-ray intensity monitors   

Monitoring the X-ray intensity can be done with a high degree of accuracy. The inline intensity monitors for X-ray energies up to 8 keV consist of three interchangeable Al-coated diamond wedges which allows for setting the diamond thickness between 10 µm and 200 µm. For the energy range 8–20 keV, two Si diodes of 100 µm and 300 µm thickness are used. The diamonds are biased and a pulsed photo-current is obtained from each X-ray pulse. The integrated charge is measured by a trans-impedance amplifier and a digital oscilloscope. In order to obtain absolute photon numbers from the diamond wedges, it is possible to calibrate them against the calibrated silicon diodes placed behind the wedges. At FemtoMAX which has a low flux compared with an FEL or a storage ring beamline, we have designed detectors to be limited by Poissonian photon statistics. This means that a reference measurement for compensation of intensity fluctuations should be set up to have the same number of detected photons as the signal. Thus, the optimal reference signal (*I*_0_) is equally as strong as the signal in the experiment. At 1.85 keV, the thinnest diamond wedge transmits 25% of the radiation, and for a 300 µm silicon diode the transmission of 20 keV radiation is 75%. If a reference signal that uses 25–75% of the incident flux is considered acceptable, intensity monitoring is available for the full range of the beamline. For weak scattering signals it would be preferable not to lose too much of the incident flux, as the signal-to-noise level then would be primarily determined by the signal. This is a limitation for tender X-rays. The lowest photon energy where less than 10% of the flux is required for the monitor is 4.4 keV.

## 5. Lasers and timing   

### 5.1. Synchronized lasers   

The FemtoMAX beamline is equipped with commercially available laser systems. The main laser is a cryo-cooled kHz Ti:sapphire amplifier (KM Laboratories Red Wyvern). It can run at a repetition rate up to 1 kHz and delivers pulses with a duration of 50 fs and pulse energy of 11 mJ at a centre wavelength of 800 nm. The reason for choosing a 1 kHz laser is amplitude stability due to the fact that CW pumped, Q-switched lasers can be used to pump the amplifiers. This is important for non-linear conversion schemes. The laser system also includes an optical parametric amplifier (OPA) (TOPAS HE from Light Conversion) with mixing stages to cover a wavelength range of 0.2–10 µm. Out of the available 800 nm laser power, 10% is split off and used for diagnostics whereas the remaining 10 W either pumps the OPA or can be used for the experiment.

### 5.2. Timing and synchronization   

Timing needs to be considered on three different timescales. On the millisecond timescale, each pulse has an identity and signals from all detectors and diagnostics are time-stamped. On faster timescales, timing pertains to the relative arrival time of the laser and X-rays (or another event from the electrons). On the 100 ps timescale the same trigger derived from the 3 GHz facility master oscillator is distributed *via* a commercial fibre system to the gun laser and to FemtoMAX. This signal provides triggers at the repetition rate of the LINAC pulses which are used for non-critical laser components, detectors and auxiliary equipment. On the femtosecond timescale, the laser oscillator is synchronized to the LINAC RF. The LINAC cavities, the gun laser and the FemtoMAX laser all get their RF from the main facility 3 GHz oscillator which is distributed on phase-stabilized rigid lines. So far the locking accuracy to the RF has been measured to be <30 fs RMS by measuring the beat note between the RF and the 39th harmonic of the laser oscillator pulse train. Locking the oscillator to the RF is necessary but not sufficient in order to obtain a low jitter in the experiments. A more direct measurement of the jitter has been carried out using non-thermal melting on InSb in a crossed-beam measurement where the complete time history is recorded in a single X-ray pulse (Lindenberg *et al.*, 2005 ▸). This method has previously been used to verify electro-optical sampling as a reliable timing tool (Cavalieri *et al.*, 2005 ▸). We found that the jitter depends strongly on the alignment of the electron beam through the bunch compressor. In the best data, 90% of the pulses appear in a 1 ps time window, but on other occasions only 25% of the pulses appeared in a 5 ps window.

One of the planned three permanently installed online jitter monitors has been developed and tested. This device measures the relative timing jitter between electrons arriving at the dump magnet and laser pulses arriving at the X-ray hutch. The first measurement was carried out by sending light from the locked laser and visible light from the bending magnet in the front-end onto fast photodiodes and subsequently filtering the diode signals into 3 GHz bandpass filters. This provides oscillating signals and their relative phase can be measured using a mixer and a fast oscilloscope. The accuracy of this scheme has been shown to be better than 200 fs when using two laser beams. However, the first measurement gave a jitter of 5 ps (RMS) while a lower jitter was measured by the direct non-thermal melting experiment, indicating that further work on this device is needed. In particular, since the visible light was weak, an avalanche photodiode (APD) was used as a detector. We believe that the randomness of the amplification process makes APDs unsuitable for this application. In the next version we will use an electron BPM signal to excite the 3 GHz bandpass filter rather than visible light from the LINAC. The second and more direct measurement of the relative timing between X-ray and laser pulses is an in-house-developed streak camera. This device will measure the relative timing for each shot which will allow for post-synchronization below the jitter level. It will also be able to compensate for the change in X-ray pulse delay during monochromator scans. The X-rays pass through the photo-cathode of the streak camera and generate photo-electrons while >90% of the X-ray flux is transmitted to the sample. UV pulses from the laser impinge on the same cathode and the delay is measured. The streak camera is placed in the hutch near the experiment. A prototype streak camera has been built and the ability to measure relative delays with an accuracy of 280 fs has been demonstrated (Enquist *et al.*, 2010 ▸). The timing streak camera will have a smaller anode slit than the prototype and is anticipated to provide a resolution of 150 fs throughout the spectral range and a time window of 10 ps when the fastest sweep speed is used. A third diagnostic which can measure the arrival time as well as the electron pulse duration is under development. The pulse shape of the X-rays will follow that of the electron bunch. Visible radiation from the bending magnet in the front-end will also follow the electron bunch shape. These dependencies enable pulse duration measurements as well as jitter measurements by means of optical cross-correlation using visible radiation from the bending magnet and 800 nm radiation from the laser (Tenishev *et al.*, 2004 ▸).

**Figure 5:**
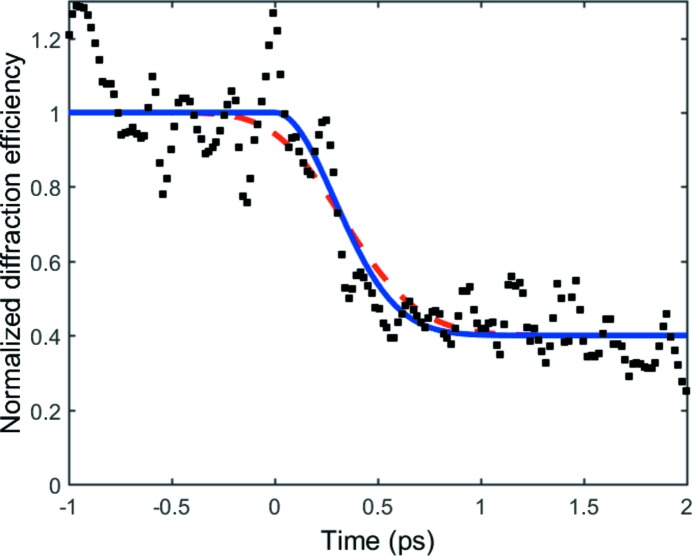
The pulse duration was estimated to be below 400 fs from a non-thermal melting experiment in crossed-beam geometry. The time axis shows delay of the probing X-ray pulse with respect to the laser pulse. The black squares are the experimental data whereas the blue curve is the model from Lindenberg *et al.* (2005 ▸). The red dashed line is the same model but convoluted with a 400 fs Gaussian curve. The noise in the experimental data is due to photon statistics. The data have been smoothed to a five-point weighted average.

The pulse duration has not yet been measured, but an indication of the pulse duration has been obtained by an experiment where InSb was non-thermally melted by an ultrashort laser pulse. The lattice loses its crystalline structure within a few hundreds femtoseconds, which manifests in a decrease of the X-ray diffraction efficiency. The experiment was carried out in a crossed-beam experiment where the temporal evolution can be captured in a single shot and the time resolution is unaffected by jitter. The data represented as black squares in Fig. 5 ▸ are compared with the simple Gaussian model that reproduced the data by Lindenberg *et al.* (2005 ▸). The blue curve represents the model response with no consideration taken of finite duration of the probing X-ray pulse, whereas the red dashed curve shows the same model response convoluted with a 400 fs pulse shape. As can be seen, the model without convolution fits best, indicating a pulse duration below 400 fs. This impression is verified by the fact that the best fit between the data and the model, in a least-squares analysis, is obtained when the model is left without convolution. When data are convoluted with a pulse, the sum of squares increases monotonically as the pulse duration is increased. Convoluting the model with a pulse of 400 fs in duration results in an increase of the sum of squares by 20%.

## 6. End-stations and sample environments   

The scope of the beamline is to provide a facility where structurally sensitive techniques can be used to measure evolving atomic structure on a femtosecond timescale. A synchronized laser will initiate a photo-induced process and subsequently the structure is measured using the pump–probe technique. Both scattering (diffraction) and X-ray spectroscopy methods will be available.

The user needs are diverse and thus a single sample environment is insufficient to cover a broad user base. A setup which could handle vacuum, liquids, atomic and molecular physics, liquid-helium temperatures *etc*. would be extremely complex and not user friendly. These requirements have been addressed by designing several user stations. The end-station for scattering and X-ray spectroscopy is intended to be mounted on a common base which has three lateral and three angular motions. This base is a precision system from Huber Diffraktionstechnik and can carry up to 600 kg if the load is centred.

For scattering in air we are providing a sample stack intended mainly for grazing-incidence measurements as well as a Kappa diffractometer. Furthermore, in-vacuum scattering environments have been developed and a low-vacuum/He-purged environment for X-ray spectroscopy is in progress. Because of the very limited amount of beam time and the high demand for accommodating many different experimental possibilities at the existing FEL facilities such as FLASH in Hamburg and LCLS in Stanford, the group of Professor Joachim Ullrich from the Max Planck Institut für Kernphysik in Heidelberg developed a highly versatile, multi-purpose user station called CAMP (Strüder *et al.*, 2010 ▸). For this station many different user groups have already developed different kinds of detection systems, like, for example, electron and ion time-of-flight spectrometers, and target sample systems, like, for example, supersonic gas beams and liquid jets, which make this chamber more and more a standard within the community working with high-peak-intensity X-ray science. In order to benefit from this development, we have built a copy of the CAMP chamber not including the PN-CCD part.

## 7. Detectors and data acquisition   

All detectors are designed to operate in vacuum, but can also be equipped with protective windows and operated in air. For in-air operation, all detectors can be mounted on a robot detector arm. The industrial robot from ABB can carry a load of 120 kg and has a position accuracy better than 80 µm. With a smaller load we have measured the reproducibility to be better than 40 µm using a laser tracker. Different detectors have been commissioned, including X-ray diodes and X-ray avalanche photodiodes as well as a 4 Mpixel Andor IKON CCD camera with a chip size of 25 mm.

We intend to include two types of two-dimensional detectors. One of them will be based on scientific CMOS technology which can be used in the range 2–7 keV although primarily designed for visible radiation. They offer high resolution <13 µm, sizes up to 50 mm while maintaining a full-frame readout speed of >50 Hz. Photon-counting detectors for tender and hard X-rays can be found at almost every scattering beamline. However, at a low-repetition-rate, short-pulse beamline, the requirements are slightly different. FemtoMAX will be equipped with a custom-built 1.2M PILATUS3 detector with the ability to count more than one photon per pixel per pulse. It is based on the instant retrigger technology which was introduced in the PILATUS3 ASIC to cope with the high photon fluxes at third-generation synchrotrons (Loeliger *et al.*, 2012 ▸). Additionally, it enables the determination of the deposited charge in each pixel by converting the current to voltage and measuring the duration for which the voltage stays over a predefined threshold. The technology has been given the name Time over Threshold (ToT). As proof of principle, a PILATUS3X 300K-W detector has been tested in this operating mode using both fluorescence targets and FemtoMAX as sources. The 300K detector features 172 µm × 172 µm pixels in a 1475 × 195 matrix. The ASIC pixels are bump-bonded to a 1 mm Si sensor. Initial simulations were carried out to determine optimal detector settings for the best possible energy resolution in ToT mode. Subsequently, the ToT of each pixel was calibrated as a function of photon energy by a large number of events in order to reduce statistical uncertainties. In the first studies a commercially available GE Titan-E X-ray tube was used. This tube was used to irradiate 13 different fluorescence targets in order to excite the *K*α lines from these targets, thus obtaining photon energies ranging from 5 to 25 keV. Each X-ray photon which is absorbed in the detector generates a cloud of electrons. The cloud may be confined in a single pixel or be spread over a number of adjacent pixels. Since only single-pixel events can be used for the calibration, the flux was kept low to achieve an average pixel occupancy of about 1% per frame. Because of the high flux of the source the exposure times could be kept in the ms range. The calibration profits from the 500 Hz frame rate of the detector since between 10000 and 100000 images were acquired for each individual target.

**Figure 6:**
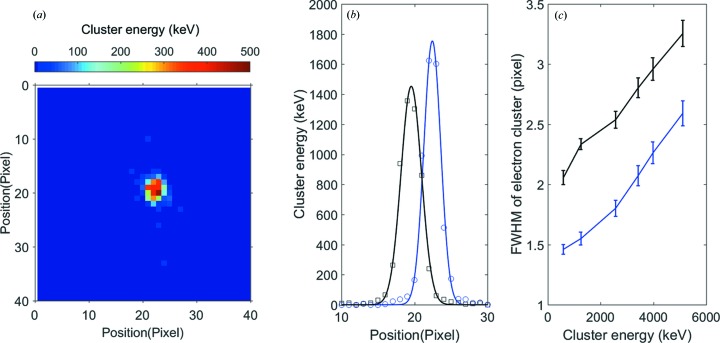
(*a*) Cluster distribution of a pulse of approximately 1000 × 5 keV photons corresponding to a total energy of 5.3 MeV focused on a single pixel. The colour scale indicates the energy deposited per pixel in keV. (*b*) Cluster profile of (*a*) along the horizontal (blue) and vertical (black) direction. The symbols are experimental points and the lines are Gaussian fits. The abscissa is the cluster energy averaged in one dimension. (*c*) The FWHM of profiles such as the one in (*b*) as a function of the total integrated energy per pulse.

Application testing was performed at FemtoMAX with 2.8 keV and 4.9 keV photons, focused on a single pixel. Different Al foils were used to attenuate the beam and create femtosecond photon pulses with known integrated photon energies between 500 keV (corresponding to 100 × 5 keV photons) and 5 MeV (corresponding to 1000 × 5 keV photons). Fig. 6 ▸ shows the spatial distribution of a 5.3 MeV pulse. Analysing the test results, each PILATUS3 ASIC pixel can provide the photon count for up to 300 keV in ToT mode with an error of <10%. Since charge sharing helps to spread the incoming charge across multiple ASIC pixels, the detector can give photon numbers up to 2.5 MeV in a single focused pulse with an error of <10%. The accuracy was evaluated by repeating the measurement a large number of times for the same number of photons per pulse. At 2.5 MeV cluster energy the spread in evaluated energy was around 200 keV FWHM, which means that the number of photons in the cluster could be calculated with an accuracy better than 90%. This development is most important to diffraction where the detected intensity is concentrated to spots. The study shows that it is possible to capture 200 × 10 keV photons for a single pulse incident on a single pixel. The maximum number of photons for any other photon energy is found from the total maximum energy which is 2 MeV incident on a single pixel.

## 8. Present status and outlook   

The FemtoMAX beamline has been built and much of the performance has been verified. Beamline diagnostics, slits, focusing optics, monochromators, harmonic rejection mirrors and the sample camera have been commissioned. The laser system performs to specification and the synchronization between the laser and the RF is better than 30 fs RMS. Installing the new short-period undulators will increase the flux significantly. The required magnetic performance has been verified at the MAX IV laboratory and installation is planned for November 2017. The parameters that have been achieved are compared with the design values in Table 7 ▸. The remaining challenges mainly include optimization of the LINAC as a driver for FemtoMAX. The repetition rate is presently limited by the existing photo-electron gun which was developed for 10 Hz. A new photo-gun which can be operated at the maximum LINAC repetition rate of 100 Hz is being manufactured. The jitter between the RF and the electron bunches can be as good as 1 ps, which is within specifications but above the 100 fs (RMS) target.

## Figures and Tables

**Table 1:** Electron bunch parameters

Parameter	Specification	Status spring 2017
Energy	3.0 GeV	3.0 GeV
Normalized RMS emittance	<10 mm mrad	<1 mm mrad
RMS energy spread	<1.5%	<1.5%
Charge per pulse	100 pC ± 10% (RMS)	100 pC ± 10%
Repetition rate	Up to 100 Hz	Up to 2 Hz
RMS pulse-to-pulse energy stability	<0.15%	<0.2%
RMS pulse-to-pulse positional stability (horizontal and vertical)	<4 µm	<10 µm
RMS pulse-to-pulse angular stability (horizontal and vertical)	<4 µrad	<10 µrad
Pulse-to-pulse intensity stability	<10% (RMS)	<±5% (max–min)
FWHM bunch length	<100 fs	<160 fs
RMS pulse-to-pulse time jitter	<1 ps	∼1 ps

**Table 2:** Source flux

	Theoretical undulator flux [photons pulse^−1^ (1% bandwidth)^−1^] 1.2 mm × 4 mm aperture at distance *d*		Measured flux [photons pulse^−1^ (1% bandwidth)^−1^] 1.2 mm × 4 mm aperture at 30 m
	Long-period undulator at 22 mm gap, *d* = 30 m	In-vacuum undulator at 2.5 mm gap, *d* = 17 m	Measured for long-period undulator. From Fig. 2 ▸, compensated for Parylene C filter and multi-layer mirror monochromator transmission
2 keV	3.5 × 10^5^ at *K* = 1.7	6 × 10^7^ at *K* = 1.9	4 × 10^5^
4 keV	1.2 × 10^5^ at *K* = 1.7	4 × 10^7^ at *K* = 0.9	1 × 10^5^
6 keV	5.7 × 10^4^ at *K* = 2.7	2.1 × 10^7^ at *K* = 1.9	5 × 10^4^
8 keV	4.0 × 10^4^ at *K* = 2.7	1.3 × 10^7^ at *K* = 1.5	3 × 10^4^
10 keV	2.5 × 10^4^ at *K* = 2.7	1.1 × 10^7^ at *K* = 1.2	2 × 10^4^
15 keV		6 × 10^6^ at *K* = 2.0	
20 keV		4 × 10^6^ at *K* = 2.1	

**Table 3:** Key parameters for the harmonic rejection mirrors

Size	120 mm × 4 mm
Material	Silicon coated with carbon
Surface type	Flat
Roughness	<5 Å RMS
Slope error	<1 µrad RMS
Mirror pair contribution to rejection ratio for fundamental *versus* third harmonic	10^4^ for fundamental photon energy 1.8–5.5 keV (calculated)
Mirror pair contribution to rejection ratio for fundamental *versus* third harmonic	10^2^ for third-harmonic photon energy 5.5–9 keV (calculated)

**Table 4:** Useable energy range of the harmonic separation prism at two different undulator gaps

Fundamental undulator energy	Minimum energy	Maximum energy
1.8 keV	3.26 keV	13.1 keV
4 keV	3.26 keV	16 keV

**Table 5:** Monochromator properties

Material	Energy range	Bandwidth (Δ*E*/*E*)
Si (111)	2.05–20 keV	<2 × 10^−4^
InSb (111)	1.72–20 keV	<4 × 10^−4^
Mo-B_4_C 44 Å	1.8–2.5 keV	<2 × 10^−2^
Ni-B_4_C 39.5 Å	2.5–8 keV	<2 × 10^−2^
Mo-B_4_C 24 Å	8–20 keV	<2 × 10^−2^

**Table 6:** Focusing mirror parameters

Size	400 mm × 25 mm
Sagittal (horizontal) bending radius	31.5 mm
Meridional (vertical) bending radius	3122–5644 m
Coating material	Rhodium
Incidence angle	2.363–3.177 mrad

**Table 7:** Key parameters for the FemtoMAX beamline

	Design	In operation July 2017
Energy (wavelength) range	1.8–20 keV (0.6–6.5 Å)	0.5–10 keV (1.2–24 Å)
Photon source	In-vacuum undulator	Temporary long-period undulator
Monochromator	Double-crystal monochromator with Si (111), InSb (111) crystals. Multi-layer mirrors (ML)	Double-crystal monochromator with Si (111), InSb (111) crystals. Multi-layer mirrors (ML)
Photons pulse^−1^ (1% bandwidth)^−1^	>1 × 10^7^ below 10 keV	>3 × 10^5^ below 4 keV; >1 × 10^5^ below 9 keV
Repetition rate	100 Hz	2 Hz
Harmonic content	<10^−3^	<10^−3^
Bandwidth	Si: Δ*E*/*E* ≃ 2 × 10^−4^ (2.5–20 keV)	Si: Δ*E*/*E* ≃ 2 × 10^−4^ (2.5–20 keV)
	InSb: Δ*E*/*E* ≃ 4 × 10^−4^ (1.8–20 keV)	InSb: Δ*E*/*E* ≃ 4 × 10^−4^ (1.8–20 keV)
	ML: Δ*E*/*E* = 0.01 (1.8–20 keV)	ML: Δ*E*/*E* = 0.01 (1.8–20 keV)
Monochomator throughput at 5 keV	>70% crystal; >50% ML	>70% crystal; >50% ML
Optics	Unfocused/rhodium-coated Si mirror, Be lenses, harmonic rejection mirror, X-ray prism	Unfocused/rhodium-coated Si mirror, Be lenses, harmonic rejection mirror, X-ray prism
Polarization	Linear	Linear
Pulse duration	<100 fs (FWHM)	<400 fs (FWHM, measured)
Synchronization	<1 ps (RMS)	<1 ps (90% of pulses within 1 ps window)
		30 fs (RMS) measured between laser and RF
Spot size on sample (H × V) (FWHM)	0.1 mm × 0.1 mm mirror	0.08 mm × 0.16 mm mirror
	100 µm × 15 µm mirror + Be lens	
Equipment	Ultrafast laser (10 mJ at 800 nm), four-circle goniometer, CCD detector, Pilatus detector, X-ray sCMOS 16 M detector	Ultrafast laser <50 fs (10 mJ at 800 nm; 0.1 mJ at 2600 nm and 530 nm), four-circle goniometer, CCD detector

## References

[bb1] Burza, M., Enquist, H., Jurgilaitis, A., Nygaard, J. & Larsson, J. (2015). *Opt. Express*, **23**, 620–627. 10.1364/OE.23.000620 25835820

[bb2] Cavalieri, A. L. *et al.* (2005). *Phys. Rev. Lett.* **94**, 114801. 10.1103/PhysRevLett.94.114801 15903864

[bb3] Caviezel, A., Staub, U., Johnson, S. L., Mariager, S. O., Möhr-Vorobeva, E., Ingold, G., Milne, C. J., Garganourakis, M., Scagnoli, V., Huang, S. W., Jia, Q. X., Cheong, S. W. & Beaud, P. (2012). *Phys. Rev. B*, **86**, 174105.

[bb4] Chin, A. H., Schoenlein, R. W., Glover, T. E., Balling, P., Leemans, W. P. & Shank, C. V. (1999). *Phys. Rev. Lett.* **83**, 336–339.

[bb5] Chollet, M. *et al.* (2015). *J. Synchrotron Rad.* **22**, 503–507. 10.1107/S1600577515005135 PMC4416667 25931060

[bb6] Daranciang, D. *et al.* (2012). *Phys. Rev. Lett.* **108**, 087601. 10.1103/PhysRevLett.108.087601 22463572

[bb7] Denecke, R., Väterlein, P., Bässler, M., Wassdahl, N., Butorin, S., Nilsson, A., Rubensson, J. E., Nordgren, J., Mårtensson, N. & Nyholm, R. (1999). *J. Electron Spectrosc. Relat. Phenom.* **101–103**, 971–977.

[bb8] Enquist, H., Navirian, H., Nüske, R., von Korff Schmising, C., Jurgilaitis, A., Herzog, M., Bargheer, M., Sondhauss, P. & Larsson, J. (2010). *Opt. Lett.* **35**, 3219–3221. 10.1364/OL.35.003219 20890339

[bb9] Gaffney, K. J. *et al.* (2005). *Phys. Rev. Lett.* **95**, 125701. 10.1103/PhysRevLett.95.125701 16197085

[bb10] Haldrup, K. *et al.* (2016). *J. Phys. Chem. B*, **120**, 1158–1168.

[bb11] Harb, M., Jurgilaitis, A., Enquist, H., Nüske, R., Schmising, C. V., Gaudin, J., Johnson, S. L., Milne, C. J., Beaud, P., Vorobeva, E., Caviezel, A., Mariager, S. O., Ingold, G. & Larsson, J. (2011). *Phys. Rev. B*, **84**, 045435.

[bb12] Ingold, G., Abela, R., Beaud, P., Johnson, S. L. & Staub, U. (2008). *Z. Kristallogr.* **223**, 292–306.

[bb13] Johnson, S. L., Beaud, P., Milne, C. J., Krasniqi, F. S., Zijlstra, E. S., Garcia, M. E., Kaiser, M., Grolimund, D., Abela, R. & Ingold, G. (2008). *Phys. Rev. Lett.* **100**, 155501. 10.1103/PhysRevLett.100.155501 18518120

[bb14] Larsson, J., Heimann, P. A., Lindenberg, A. M., Schuck, P. J., Bucksbaum, P. H., Lee, R. W., Padmore, H. A., Wark, J. S. & Falcone, R. W. (1998). *Appl. Phys. A*, **66**, 587–591.

[bb16] Lindenberg, A. M., Kang, I., Johnson, S. L., Missalla, T., Heimann, P. A., Chang, Z., Larsson, J., Bucksbaum, P. H., Kapteyn, H. C., Padmore, H. A., Lee, R. W., Wark, J. S. & Falcone, R. W. (2000). *Phys. Rev. Lett.* **84**, 111–114. 10.1103/PhysRevLett.84.111 11015847

[bb17] Lindenberg, A. M. *et al.* (2005). *Science*, **308**, 392–395. 10.1126/science.1107996 15831753

[bb15] Lindenberg, A. M. *et al.* (2008). *Phys. Rev. Lett.* **100**, 135502. 10.1103/PhysRevLett.100.135502 18517965

[bb18] Loeliger, T., Bronnimann, C., Donath, T., Schneebeli, M., Schnyder, R. & Trub, P. (2012). *2012 IEEE Nuclear Science Symposium and Medical Imaging Conference Record (NSS/MIC)*, pp. 610–615. IEEE.

[bb19] Mariager, S. O., Pressacco, F., Ingold, G., Caviezel, A., Möhr-Vorobeva, E., Beaud, P., Johnson, S. L., Milne, C. J., Mancini, E., Moyerman, S., Fullerton, E. E., Feidenhans’l, R., Back, C. H. & Quitmann, C. (2012). *Phys. Rev. Lett.* **108**, 087201. 10.1103/PhysRevLett.108.087201 22463562

[bb20] Nicoul, M., Shymanovich, U., Kahle, S., Caughey, T., Sampat, D., Sokolowski-Tinten, K. & von der Linde, D. (2005). *J. Phys. Conf. Ser.* **21**, 207–210.

[bb21] Rischel, C., Rousse, A., Uschmann, I., Albouy, P. A., Geindre, J. P., Audebert, P., Gauthier, J. C., Fröster, E., Martin, J. L. & Antonetti, A. (1997). *Nature (London)*, **390**, 490–492.

[bb22] Rose-Petruck, C., Jimenez, R., Guo, T., Cavalleri, A., Siders, C. W., Rksi, F., Squier, J. A., Walker, B. C., Wilson, K. R. & Barty, C. P. J. (1999). *Nature (London)*, **398**, 310–312.

[bb23] Rousse, A., Rischel, C., Fourmaux, S., Uschmann, I., Sebban, S., Grillon, G., Balcou, P., Förster, E., Geindre, J. P., Audebert, P., Gauthier, J. C. & Hulin, D. (2001). *Nature (London)*, **410**, 65–68. 10.1038/35065045 11242040

[bb24] Schoenlein, R. W., Chattopadhyay, S., Chong, H. H. W., Glover, T. E., Heimann, P. A., Shank, C. V., Zholents, A. A. & Zolotorev, M. S. (2000). *Science*, **287**, 2237–2240. 10.1126/science.287.5461.2237 10731140

[bb25] Sokolowski-Tinten, K., Blome, C., Blums, J., Cavalleri, A., Dietrich, C., Tarasevitch, A., Uschmann, I., Förster, E., Kammler, M., Horn-von-Hoegen, M. & von der Linde, D. (2003). *Nature (London)*, **422**, 287–289. 10.1038/nature01490 12646915

[bb26] Sokolowski-Tinten, K., Blome, C., Dietrich, C., Tarasevitch, A., Horn von Hoegen, M., von der Linde, D., Cavalleri, A., Squier, J. & Kammler, M. (2001). *Phys. Rev. Lett.* **87**, 225701. 10.1103/PhysRevLett.87.225701 11736408

[bb27] Srajer, V., Teng, T. Y., Ursby, T., Pradervand, C., Ren, Z., Adachi, S., Schildkamp, W., Bourgeois, D., Wulff, M. & Moffat, K. (1996). *Science*, **274**, 1726–1729. 10.1126/science.274.5293.1726 8939867

[bb28] Strüder, L. *et al.* (2010). *Nucl. Instrum. Methods Phys. Res. A*, **614**, 483–496.

[bb29] Tanaka, T. & Kitamura, H. (2001). *J. Synchrotron Rad.* **8**, 1221–1228. 10.1107/s090904950101425x 11679776

[bb30] Tenishev, V., Avdeichikov, V., Persson, A. & Larsson, J. (2004). *Meas. Sci. Technol.* **15**, 1762–1767.

[bb31] Thorin, S. A. J., Curbis, F., Eriksson, M., Karlberg, O., Kumbaro, D., Mansten, E., Olsson, D. & Werin, S. (2014). *27th International Linear Accelerator Conference (LINAC 14)*, pp. 400–403. Geneva: JACoW.

[bb32] Thorin, S. E. M., Werin, S., Angal-Khalinin, D., McKenzie, J., Militsyn, B. & Williams, P. (2010). *32nd International Free Electron Laser Conference (FEL 2010)* pp. 471–474. Malmö: JACoW.

[bb33] Trigo, M. *et al.* (2013). *Nat. Phys.* **9**, 790–794.

[bb34] Werin, S., Thorin, S., Eriksson, M. & Larsson, J. (2009). *Nucl. Instrum. Methods Phys. Res. A*, **601**, 98–107.

